# Bis(benzo-15-crown-5-κ^5^ *O*)barium tetrakis­(isothio­cyanato-κ*N*)cobaltate(II)

**DOI:** 10.1107/S1600536809053471

**Published:** 2009-12-16

**Authors:** Zhiqiang Cao, Kai Liu, Meiju Niu, Daqi Wang

**Affiliations:** a College of Chemistry and Chemical Engineering, Liaocheng University, Shandong 252059, People’s Republic of China

## Abstract

In the title compound, [Ba(C_14_H_20_O_5_)_2_][Co(NCS)_4_], the Ba^II^ and Co^II^ ions are situated on twofold rotational axes, so asymmetric unit contains half each of the complex cations and anions. The Co^II^ ion is coordinated by four N atoms [Co—N 1.83 (2), 1.95 (3) Å] in a distorted tetra­hedral geometry. The Ba^II^ ion is coordinated by ten O atoms [Ba—O 2.766 (19)–2.859 (19) Å] from two benzo-15-crown-5 ligands in a sandwich-like configuration.

## Related literature

For related structures, see: Drew *et al.* (1983 ▶); Owen (1983 ▶); Nunez & Rogers (1993 ▶).
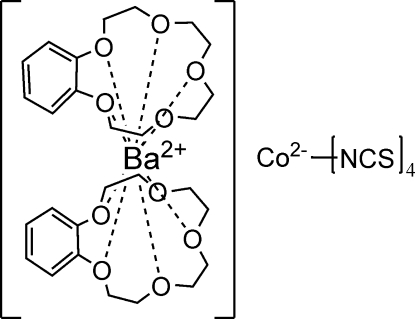


## Experimental

### 

#### Crystal data

[Ba(C_14_H_20_O_5_)_2_][Co(NCS)_4_]
*M* *_r_* = 965.19
Trigonal, 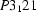

*a* = 12.5772 (13) Å
*c* = 23.758 (3) Å
*V* = 3254.7 (6) Å^3^
*Z* = 3
Mo *K*α radiation
μ = 1.53 mm^−1^
*T* = 298 K
0.43 × 0.40 × 0.28 mm

#### Data collection

Bruker SMART CCD area-detector diffractometer
Absorption correction: multi-scan (*SADABS*; Sheldrick, 1996 ▶) *T*_min_ = 0.559, *T*_max_ = 0.674
14488 measured reflections
3786 independent reflections
2749 reflections with *I* > 2σ(*I*)
*R*_int_ = 0.059

#### Refinement

*R*[*F* ^2^ > 2σ(*F* ^2^)] = 0.068
*wR*(*F* ^2^) = 0.196
*S* = 1.00
3786 reflections
236 parameters
1 restraint
H-atom parameters constrained
Δρ_max_ = 1.68 e Å^−3^
Δρ_min_ = −1.38 e Å^−3^
Absolute structure: Flack (1983 ▶), 1623 Friedel pairs
Flack parameter: −0.01 (12)

### 

Data collection: *SMART* (Siemens, 1996 ▶); cell refinement: *SAINT* (Siemens, 1996 ▶); data reduction: *SAINT*; program(s) used to solve structure: *SHELXS97* (Sheldrick, 2008 ▶); program(s) used to refine structure: *SHELXL97* (Sheldrick, 2008 ▶); molecular graphics: *SHELXTL* (Sheldrick, 2008 ▶); software used to prepare material for publication: *SHELXTL*.

## Supplementary Material

Crystal structure: contains datablocks I, global. DOI: 10.1107/S1600536809053471/cv2669sup1.cif

Structure factors: contains datablocks I. DOI: 10.1107/S1600536809053471/cv2669Isup2.hkl

Additional supplementary materials:  crystallographic information; 3D view; checkCIF report

## Figures and Tables

**Fig. 1.**
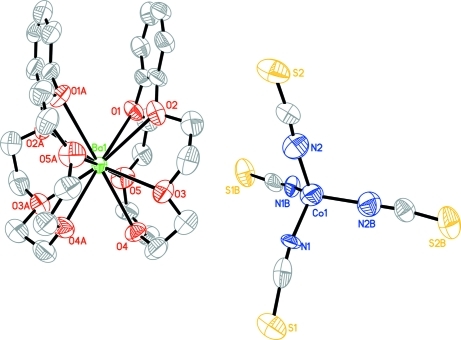
The molecular structure of (I), showing 30% probability displacement ellipsoids and atomic numbering for non-C atoms [symmetry codes: (A) -x, -x + y, 4/3 - z; (B)1 + x-y,2 - y, 5/3 - z]. H atoms omitted for clarity.

## supplementary crystallographic information

### Comment

In continuation of structural study of benzo-15-crown-5 ligands and their barium(II) complexes (Nunez *et al.*, 1993), we report here the synthesis and crystal structure of the title compound, (I).

In (I) (Fig. 1), the Ba^II^ ions are sandwiched between the 15-crown-5 rings from two ligands (Owen, 1983). The Co^II^ ions are coordinated by four N atoms from the thiocyanate ligands in a distorted tetrahedal geometry. The Ba—O and Co—N coordinating bond lengths are comparable with those observed in other Ba^II^ (Nunez *et al.*, 1993) and Co^II^ complexes (Drew *et al.*, 1983).

### Experimental

Potassium thiocyanate (1 mmol, 97.18 mg) and cobalt(II) dichloride (0.25 mmol, 59.49 mg) were dissolved and stirred at 293 K in H_2_O solution (10 ml) for 0.5 h and then added dropwise to a 1,2-dichloroethane solution (10 ml) of benzo-15-crown-5 (0.5 mmol,134.16 mg). The mixture was then stirred at 293 K for 0.5 h. An aqueous solution (2 ml) of barium(II) chloride (0.25 mmol, 52.07 mg) was then added dropwise and the mixture stirred for another 5 h. The lower clear liquid was held at room temperature for about one week, whereupon blue square block crystal suitable for X-ray diffraction analysis were obtained.

### Refinement

All H atoms were placed in geometrically idealized positions (C—H 0.93-0.97 Å) and treated as riding on their parent atoms, with *U*_iso_(H) = 1.2*U*_eq_(C).

### Crystal data

**Table:**

[Ba(C_14_H_20_O_5_)_2_][Co(NCS)_4_]	*D*_x_ = 1.477 Mg m^−^^3^
*M**_r_* = 965.19	Mo *K*α radiation, λ = 0.71073 Å
Trigonal, *P*3_1_21	Cell parameters from 2944 reflections
*a* = 12.5772 (13) Å	θ = 2.5–18.7°
*c* = 23.758 (3) Å	µ = 1.53 mm^−^^1^
*V* = 3254.7 (6) Å^3^	*T* = 298 K
*Z* = 3	Block, blue
*F*(000) = 1461	0.43 × 0.40 × 0.28 mm

### Data collection

**Table:**

Bruker SMART CCD area-detector diffractometer	3786 independent reflections
Radiation source: fine-focus sealed tube	2749 reflections with *I* > 2σ(*I*)
graphite	*R*_int_ = 0.059
phi and ω scans	θ_max_ = 25.0°, θ_min_ = 1.9°
Absorption correction: multi-scan (*SADABS*; Sheldrick, 1996)	*h* = −10→14
*T*_min_ = 0.559, *T*_max_ = 0.674	*k* = −14→14
14488 measured reflections	*l* = −28→26

### Refinement

**Table:**

Refinement on *F*^2^	Secondary atom site location: difference Fourier map
Least-squares matrix: full	Hydrogen site location: inferred from neighbouring sites
*R*[*F*^2^ > 2σ(*F*^2^)] = 0.068	H-atom parameters constrained
*wR*(*F*^2^) = 0.196	*w* = 1/[σ^2^(*F*_o_^2^) + (0.1148*P*)^2^] where *P* = (*F*_o_^2^ + 2*F*_c_^2^)/3
*S* = 1.00	(Δ/σ)_max_ = 0.001
3786 reflections	Δρ_max_ = 1.68 e Å^−^^3^
236 parameters	Δρ_min_ = −1.38 e Å^−^^3^
1 restraint	Absolute structure: Flack (1983), 1623 Friedel pairs
Primary atom site location: structure-invariant direct methods	Flack parameter: −0.01 (12)

### Fractional atomic coordinates and isotropic or equivalent isotropic displacement parameters (Å^2^)

**Table:**

	*x*	*y*	*z*	*U*_iso_*/*U*_eq_	
Ba1	0.0000	0.58989 (14)	0.6667	0.0538 (6)	
Co1	0.5798 (5)	1.0000	0.8333	0.1076 (17)	
N1	0.553 (2)	1.113 (2)	0.7970 (9)	0.069 (6)	
N2	0.615 (3)	0.900 (3)	0.7820 (13)	0.119 (9)	
O1	−0.0086 (19)	0.409 (2)	0.7422 (9)	0.087 (6)	
O2	0.1988 (19)	0.561 (2)	0.6987 (9)	0.097 (7)	
O3	0.222 (2)	0.794 (2)	0.6989 (9)	0.102 (6)	
O4	0.002 (2)	0.773 (2)	0.7356 (9)	0.096 (6)	
O5	−0.130 (2)	0.530 (2)	0.7666 (9)	0.111 (7)	
S1	0.5021 (12)	1.3067 (13)	0.7753 (4)	0.127 (4)	
S2	0.6535 (14)	0.7424 (14)	0.7203 (5)	0.147 (5)	
C1	0.068 (3)	0.364 (3)	0.7324 (14)	0.094 (9)	
C2	0.028 (4)	0.241 (4)	0.7438 (16)	0.118 (13)	
H2	−0.0487	0.1890	0.7594	0.141*	
C3	0.110 (5)	0.201 (4)	0.7304 (16)	0.126 (14)	
H3	0.0906	0.1213	0.7385	0.151*	
C4	0.218 (4)	0.281 (4)	0.7053 (15)	0.108 (11)	
H4	0.2689	0.2499	0.6954	0.129*	
C5	0.260 (4)	0.403 (4)	0.6928 (14)	0.111 (12)	
H5	0.3340	0.4523	0.6743	0.133*	
C6	0.182 (3)	0.445 (4)	0.7105 (13)	0.090 (9)	
C7	0.300 (4)	0.651 (4)	0.7258 (19)	0.124 (13)	
H7A	0.3695	0.6377	0.7202	0.149*	
H7B	0.2846	0.6482	0.7659	0.149*	
C8	0.329 (4)	0.774 (4)	0.7017 (17)	0.131 (13)	
H8A	0.3927	0.8380	0.7245	0.157*	
H8B	0.3617	0.7808	0.6640	0.157*	
C9	0.228 (4)	0.877 (4)	0.7402 (16)	0.119 (13)	
H9A	0.3014	0.9570	0.7355	0.143*	
H9B	0.2281	0.8470	0.7777	0.143*	
C10	0.113 (4)	0.884 (4)	0.7295 (17)	0.121 (13)	
H10A	0.1125	0.9438	0.7553	0.145*	
H10B	0.1171	0.9148	0.6916	0.145*	
C11	−0.059 (4)	0.743 (3)	0.7883 (14)	0.106 (11)	
H11A	−0.0932	0.7958	0.7956	0.128*	
H11B	−0.0013	0.7552	0.8182	0.128*	
C12	−0.160 (3)	0.610 (4)	0.7861 (14)	0.101 (11)	
H12A	−0.1920	0.5849	0.8239	0.121*	
H12B	−0.2261	0.6048	0.7631	0.121*	
C13	−0.126 (3)	0.453 (4)	0.8075 (13)	0.102 (10)	
H13A	−0.2086	0.3862	0.8149	0.122*	
H13B	−0.0935	0.4983	0.8422	0.122*	
C14	−0.047 (5)	0.401 (4)	0.7888 (16)	0.128 (14)	
H14A	0.0241	0.4360	0.8134	0.154*	
H14B	−0.0929	0.3136	0.7974	0.154*	
C15	0.529 (3)	1.183 (2)	0.7886 (11)	0.098 (11)	
C16	0.639 (3)	0.835 (3)	0.7554 (14)	0.094 (9)	

### Atomic displacement parameters (Å^2^)

**Table:**

	*U*^11^	*U*^22^	*U*^33^	*U*^12^	*U*^13^	*U*^23^
Ba1	0.0643 (13)	0.0576 (9)	0.0417 (9)	0.0321 (6)	−0.0018 (10)	−0.0009 (5)
Co1	0.101 (3)	0.091 (4)	0.128 (5)	0.045 (2)	−0.003 (2)	−0.007 (4)
N1	0.088 (16)	0.076 (14)	0.078 (14)	0.067 (13)	−0.009 (11)	−0.004 (11)
N2	0.13 (2)	0.12 (2)	0.11 (2)	0.06 (2)	0.010 (17)	−0.002 (18)
O1	0.094 (14)	0.119 (17)	0.072 (12)	0.070 (14)	0.009 (11)	0.025 (12)
O2	0.081 (14)	0.130 (19)	0.082 (14)	0.053 (14)	−0.005 (11)	0.012 (13)
O3	0.104 (15)	0.099 (16)	0.094 (14)	0.044 (14)	−0.009 (12)	−0.020 (13)
O4	0.116 (18)	0.092 (15)	0.087 (14)	0.059 (15)	0.003 (13)	−0.021 (11)
O5	0.133 (19)	0.126 (18)	0.083 (13)	0.070 (17)	0.038 (14)	0.007 (14)
S1	0.159 (9)	0.182 (11)	0.091 (5)	0.123 (8)	0.024 (6)	0.023 (7)
S2	0.170 (11)	0.177 (12)	0.150 (9)	0.129 (10)	0.032 (9)	0.023 (9)
C1	0.12 (3)	0.11 (3)	0.08 (2)	0.08 (2)	−0.021 (19)	−0.01 (2)
C2	0.14 (3)	0.13 (4)	0.10 (3)	0.08 (3)	−0.03 (2)	0.00 (2)
C3	0.15 (4)	0.14 (4)	0.10 (3)	0.08 (4)	−0.03 (3)	−0.01 (3)
C4	0.14 (3)	0.14 (3)	0.10 (2)	0.11 (3)	−0.02 (2)	−0.02 (2)
C5	0.13 (3)	0.14 (3)	0.10 (2)	0.09 (3)	−0.03 (2)	−0.01 (2)
C6	0.11 (3)	0.13 (3)	0.081 (18)	0.09 (3)	−0.009 (18)	−0.004 (19)
C7	0.12 (3)	0.13 (3)	0.12 (3)	0.06 (3)	−0.01 (3)	0.00 (3)
C8	0.11 (3)	0.13 (3)	0.11 (3)	0.03 (3)	−0.01 (2)	−0.01 (3)
C9	0.12 (3)	0.11 (3)	0.11 (3)	0.04 (2)	0.00 (2)	−0.01 (2)
C10	0.14 (4)	0.10 (3)	0.12 (3)	0.05 (2)	−0.01 (2)	−0.03 (2)
C11	0.13 (3)	0.13 (3)	0.09 (2)	0.09 (3)	0.01 (2)	−0.010 (19)
C12	0.12 (3)	0.13 (3)	0.09 (2)	0.09 (3)	0.035 (19)	0.01 (2)
C13	0.11 (2)	0.14 (3)	0.061 (17)	0.06 (2)	0.019 (17)	0.02 (2)
C14	0.16 (4)	0.13 (3)	0.09 (3)	0.07 (3)	0.00 (3)	0.01 (3)
C15	0.062 (17)	0.15 (3)	0.056 (16)	0.031 (19)	0.008 (14)	−0.024 (19)
C16	0.10 (2)	0.11 (2)	0.10 (2)	0.063 (19)	0.011 (18)	0.018 (18)

### Geometric parameters (Å, °)

**Table:**

Ba1—O5	2.766 (19)	C1—C2	1.39 (5)
Ba1—O5^i^	2.766 (19)	C2—C3	1.39 (6)
Ba1—O3	2.79 (2)	C2—H2	0.9300
Ba1—O3^i^	2.79 (2)	C3—C4	1.36 (5)
Ba1—O2	2.81 (2)	C3—H3	0.9300
Ba1—O2^i^	2.81 (2)	C4—C5	1.38 (5)
Ba1—O4	2.816 (18)	C4—H4	0.9300
Ba1—O4^i^	2.816 (18)	C5—C6	1.40 (4)
Ba1—O1	2.859 (19)	C5—H5	0.9300
Ba1—O1^i^	2.859 (19)	C7—C8	1.51 (6)
Co1—N1	1.834 (19)	C7—H7A	0.9700
Co1—N1^ii^	1.83 (2)	C7—H7B	0.9700
Co1—N2	1.95 (3)	C8—H8A	0.9700
Co1—N2^ii^	1.95 (3)	C8—H8B	0.9700
N1—C15	1.07 (3)	C9—C10	1.52 (5)
N2—C16	1.19 (4)	C9—H9A	0.9700
O1—C14	1.19 (4)	C9—H9B	0.9700
O1—C1	1.36 (4)	C10—H10A	0.9700
O2—C7	1.37 (4)	C10—H10B	0.9700
O2—C6	1.39 (4)	C11—C12	1.52 (5)
O3—C9	1.41 (4)	C11—H11A	0.9700
O3—C8	1.49 (5)	C11—H11B	0.9700
O4—C10	1.40 (5)	C12—H12A	0.9700
O4—C11	1.42 (4)	C12—H12B	0.9700
O5—C12	1.32 (4)	C13—C14	1.51 (5)
O5—C13	1.40 (4)	C13—H13A	0.9700
S1—C15	1.79 (2)	C13—H13B	0.9700
S2—C16	1.51 (4)	C14—H14A	0.9700
C1—C6	1.38 (5)	C14—H14B	0.9700
			
O5—Ba1—O5^i^	177.1 (11)	O1—C1—C6	117 (3)
O5—Ba1—O3	101.4 (7)	O1—C1—C2	120 (4)
O5^i^—Ba1—O3	77.3 (7)	C6—C1—C2	124 (3)
O5—Ba1—O3^i^	77.3 (7)	C3—C2—C1	116 (4)
O5^i^—Ba1—O3^i^	101.4 (7)	C3—C2—H2	122.0
O3—Ba1—O3^i^	130.4 (10)	C1—C2—H2	122.0
O5—Ba1—O2	100.2 (7)	C4—C3—C2	119 (4)
O5^i^—Ba1—O2	81.5 (7)	C4—C3—H3	120.5
O3—Ba1—O2	59.9 (8)	C2—C3—H3	120.5
O3^i^—Ba1—O2	169.6 (7)	C3—C4—C5	127 (4)
O5—Ba1—O2^i^	81.5 (7)	C3—C4—H4	116.7
O5^i^—Ba1—O2^i^	100.2 (7)	C5—C4—H4	116.7
O3—Ba1—O2^i^	169.6 (7)	C4—C5—C6	114 (4)
O3^i^—Ba1—O2^i^	59.9 (8)	C4—C5—H5	123.0
O2—Ba1—O2^i^	109.9 (11)	C6—C5—H5	123.0
O5—Ba1—O4	59.0 (7)	C1—C6—O2	114 (3)
O5^i^—Ba1—O4	118.2 (8)	C1—C6—C5	120 (3)
O3—Ba1—O4	59.4 (7)	O2—C6—C5	125 (4)
O3^i^—Ba1—O4	80.0 (7)	O2—C7—C8	108 (3)
O2—Ba1—O4	107.6 (7)	O2—C7—H7A	110.1
O2^i^—Ba1—O4	129.2 (7)	C8—C7—H7A	110.1
O5—Ba1—O4^i^	118.2 (8)	O2—C7—H7B	110.1
O5^i^—Ba1—O4^i^	59.0 (7)	C8—C7—H7B	110.1
O3—Ba1—O4^i^	80.0 (7)	H7A—C7—H7B	108.4
O3^i^—Ba1—O4^i^	59.4 (7)	O3—C8—C7	115 (3)
O2—Ba1—O4^i^	129.2 (7)	O3—C8—H8A	108.6
O2^i^—Ba1—O4^i^	107.6 (7)	C7—C8—H8A	108.6
O4—Ba1—O4^i^	71.1 (9)	O3—C8—H8B	108.6
O5—Ba1—O1	57.6 (7)	C7—C8—H8B	108.6
O5^i^—Ba1—O1	125.1 (7)	H8A—C8—H8B	107.6
O3—Ba1—O1	100.5 (6)	O3—C9—C10	103 (3)
O3^i^—Ba1—O1	118.0 (6)	O3—C9—H9A	111.1
O2—Ba1—O1	53.7 (6)	C10—C9—H9A	111.1
O2^i^—Ba1—O1	72.4 (7)	O3—C9—H9B	111.1
O4—Ba1—O1	105.6 (6)	C10—C9—H9B	111.1
O4^i^—Ba1—O1	175.9 (7)	H9A—C9—H9B	109.1
O5—Ba1—O1^i^	125.1 (7)	O4—C10—C9	115 (3)
O5^i^—Ba1—O1^i^	57.6 (7)	O4—C10—H10A	108.4
O3—Ba1—O1^i^	118.0 (6)	C9—C10—H10A	108.4
O3^i^—Ba1—O1^i^	100.5 (6)	O4—C10—H10B	108.4
O2—Ba1—O1^i^	72.4 (7)	C9—C10—H10B	108.4
O2^i^—Ba1—O1^i^	53.7 (6)	H10A—C10—H10B	107.5
O4—Ba1—O1^i^	175.9 (7)	O4—C11—C12	108 (3)
O4^i^—Ba1—O1^i^	105.6 (6)	O4—C11—H11A	110.1
O1—Ba1—O1^i^	77.9 (9)	C12—C11—H11A	110.1
N1—Co1—N1^ii^	110.4 (15)	O4—C11—H11B	110.1
N1—Co1—N2	113.2 (12)	C12—C11—H11B	110.1
N1^ii^—Co1—N2	103.5 (11)	H11A—C11—H11B	108.4
N1—Co1—N2^ii^	103.5 (11)	O5—C12—C11	117 (3)
N1^ii^—Co1—N2^ii^	113.2 (12)	O5—C12—H12A	108.1
N2—Co1—N2^ii^	113.2 (19)	C11—C12—H12A	108.1
C15—N1—Co1	162 (3)	O5—C12—H12B	108.1
C16—N2—Co1	173 (3)	C11—C12—H12B	108.1
C14—O1—C1	118 (3)	H12A—C12—H12B	107.3
C14—O1—Ba1	120 (3)	O5—C13—C14	111 (3)
C1—O1—Ba1	118.2 (19)	O5—C13—H13A	109.4
C7—O2—C6	111 (3)	C14—C13—H13A	109.4
C7—O2—Ba1	123 (2)	O5—C13—H13B	109.4
C6—O2—Ba1	120.6 (19)	C14—C13—H13B	109.4
C9—O3—C8	112 (3)	H13A—C13—H13B	108.0
C9—O3—Ba1	122 (2)	O1—C14—C13	124 (4)
C8—O3—Ba1	115 (2)	O1—C14—H14A	106.2
C10—O4—C11	119 (3)	C13—C14—H14A	106.2
C10—O4—Ba1	110.8 (19)	O1—C14—H14B	106.2
C11—O4—Ba1	120.9 (19)	C13—C14—H14B	106.2
C12—O5—C13	114 (2)	H14A—C14—H14B	106.4
C12—O5—Ba1	117 (2)	N1—C15—S1	176 (3)
C13—O5—Ba1	124 (2)	N2—C16—S2	173 (3)
			
N1^ii^—Co1—N1—C15	55 (8)	O5—Ba1—O4—C11	4(2)
N2—Co1—N1—C15	171 (8)	O5^i^—Ba1—O4—C11	−178 (2)
N2^ii^—Co1—N1—C15	−66 (9)	O3—Ba1—O4—C11	−125 (3)
N1—Co1—N2—C16	174 (26)	O3^i^—Ba1—O4—C11	85 (2)
N1^ii^—Co1—N2—C16	−66 (26)	O2—Ba1—O4—C11	−88 (2)
N2^ii^—Co1—N2—C16	57 (26)	O2^i^—Ba1—O4—C11	48 (3)
O5—Ba1—O1—C14	−13 (3)	O4^i^—Ba1—O4—C11	146 (3)
O5^i^—Ba1—O1—C14	166 (3)	O1—Ba1—O4—C11	−32 (2)
O3—Ba1—O1—C14	84 (3)	O1^i^—Ba1—O4—C11	−178 (9)
O3^i^—Ba1—O1—C14	−64 (3)	O5^i^—Ba1—O5—C12	2(2)
O2—Ba1—O1—C14	123 (3)	O3—Ba1—O5—C12	66 (2)
O2^i^—Ba1—O1—C14	−104 (3)	O3^i^—Ba1—O5—C12	−63 (2)
O4—Ba1—O1—C14	23 (3)	O2—Ba1—O5—C12	127 (2)
O4^i^—Ba1—O1—C14	−13 (12)	O2^i^—Ba1—O5—C12	−124 (3)
O1^i^—Ba1—O1—C14	−160 (4)	O4—Ba1—O5—C12	23 (2)
O5—Ba1—O1—C1	−170 (2)	O4^i^—Ba1—O5—C12	−18 (3)
O5^i^—Ba1—O1—C1	8(3)	O1—Ba1—O5—C12	162 (3)
O3—Ba1—O1—C1	−74 (2)	O1^i^—Ba1—O5—C12	−157 (2)
O3^i^—Ba1—O1—C1	139 (2)	O5^i^—Ba1—O5—C13	−152 (3)
O2—Ba1—O1—C1	−34 (2)	O3—Ba1—O5—C13	−88 (3)
O2^i^—Ba1—O1—C1	99 (2)	O3^i^—Ba1—O5—C13	143 (3)
O4—Ba1—O1—C1	−134 (2)	O2—Ba1—O5—C13	−27 (3)
O4^i^—Ba1—O1—C1	−170 (9)	O2^i^—Ba1—O5—C13	82 (3)
O1^i^—Ba1—O1—C1	43 (2)	O4—Ba1—O5—C13	−131 (3)
O5—Ba1—O2—C7	−83 (3)	O4^i^—Ba1—O5—C13	−173 (2)
O5^i^—Ba1—O2—C7	94 (3)	O1—Ba1—O5—C13	7(2)
O3—Ba1—O2—C7	14 (3)	O1^i^—Ba1—O5—C13	49 (3)
O3^i^—Ba1—O2—C7	−159 (4)	C14—O1—C1—C6	−123 (4)
O2^i^—Ba1—O2—C7	−168 (3)	Ba1—O1—C1—C6	35 (4)
O4—Ba1—O2—C7	−23 (3)	C14—O1—C1—C2	58 (5)
O4^i^—Ba1—O2—C7	57 (3)	Ba1—O1—C1—C2	−144 (3)
O1—Ba1—O2—C7	−119 (3)	O1—C1—C2—C3	177 (3)
O1^i^—Ba1—O2—C7	153 (3)	C6—C1—C2—C3	−2(5)
O5—Ba1—O2—C6	69 (2)	C1—C2—C3—C4	−3(5)
O5^i^—Ba1—O2—C6	−114 (2)	C2—C3—C4—C5	3(6)
O3—Ba1—O2—C6	166 (2)	C3—C4—C5—C6	2(5)
O3^i^—Ba1—O2—C6	−7(5)	O1—C1—C6—O2	−3(4)
O2^i^—Ba1—O2—C6	−15.7 (18)	C2—C1—C6—O2	176 (3)
O4—Ba1—O2—C6	129 (2)	O1—C1—C6—C5	−172 (3)
O4^i^—Ba1—O2—C6	−151.0 (19)	C2—C1—C6—C5	7(5)
O1—Ba1—O2—C6	32.7 (19)	C7—O2—C6—C1	124 (3)
O1^i^—Ba1—O2—C6	−55 (2)	Ba1—O2—C6—C1	−31 (3)
O5—Ba1—O3—C9	−33 (3)	C7—O2—C6—C5	−67 (4)
O5^i^—Ba1—O3—C9	144 (3)	Ba1—O2—C6—C5	137 (3)
O3^i^—Ba1—O3—C9	50 (2)	C4—C5—C6—C1	−7(5)
O2—Ba1—O3—C9	−128 (3)	C4—C5—C6—O2	−174 (3)
O2^i^—Ba1—O3—C9	−138 (4)	C6—O2—C7—C8	168 (3)
O4—Ba1—O3—C9	10 (2)	Ba1—O2—C7—C8	−37 (4)
O4^i^—Ba1—O3—C9	84 (3)	C9—O3—C8—C7	106 (4)
O1—Ba1—O3—C9	−92 (2)	Ba1—O3—C8—C7	−40 (4)
O1^i^—Ba1—O3—C9	−173 (2)	O2—C7—C8—O3	50 (5)
O5—Ba1—O3—C8	109 (2)	C8—O3—C9—C10	180 (3)
O5^i^—Ba1—O3—C8	−73 (2)	Ba1—O3—C9—C10	−37 (4)
O3^i^—Ba1—O3—C8	−168 (2)	C11—O4—C10—C9	92 (4)
O2—Ba1—O3—C8	14 (2)	Ba1—O4—C10—C9	−55 (4)
O2^i^—Ba1—O3—C8	4(5)	O3—C9—C10—O4	61 (4)
O4—Ba1—O3—C8	153 (2)	C10—O4—C11—C12	−169 (3)
O4^i^—Ba1—O3—C8	−134 (2)	Ba1—O4—C11—C12	−25 (4)
O1—Ba1—O3—C8	51 (2)	C13—O5—C12—C11	109 (3)
O1^i^—Ba1—O3—C8	−31 (2)	Ba1—O5—C12—C11	−48 (4)
O5—Ba1—O4—C10	151 (2)	O4—C11—C12—O5	48 (5)
O5^i^—Ba1—O4—C10	−31 (2)	C12—O5—C13—C14	−158 (3)
O3—Ba1—O4—C10	22 (2)	Ba1—O5—C13—C14	−3(4)
O3^i^—Ba1—O4—C10	−128 (2)	C1—O1—C14—C13	176 (4)
O2—Ba1—O4—C10	59 (2)	Ba1—O1—C14—C13	19 (6)
O2^i^—Ba1—O4—C10	−165 (2)	O5—C13—C14—O1	−11 (6)
O4^i^—Ba1—O4—C10	−67 (2)	Co1—N1—C15—S1	95 (36)
O1—Ba1—O4—C10	115 (2)	Co1—N2—C16—S2	105 (33)
O1^i^—Ba1—O4—C10	−31 (11)		

Symmetry codes: (i) −*x*, −*x*+*y*, −*z*+4/3; (ii) *x*−*y*+1, −*y*+2, −*z*+5/3.

### Hydrogen-bond geometry (Å, °)

**Table:**

*D*—H···*A*	*D*—H	H···*A*	*D*···*A*	*D*—H···*A*
C12—H12B···S2^iii^	0.97	2.99	3.83 (4)	145
C8—H8B···S1^iv^	0.97	2.97	3.93 (4)	170

Symmetry codes: (iii) *x*−1, *y*, *z*; (iv) −*x*+1, −*x*+*y*, −*z*+4/3.
